# Efficacy and mechanism of long-snake moxibustion for treating insomnia in breast cancer survivors: study protocol for a randomized controlled trial

**DOI:** 10.3389/fneur.2025.1524412

**Published:** 2025-04-30

**Authors:** Cuicui Gong, Huakang Li, Qiang Li, Pengxuan Gu, Qi Xiao, Yunjing Jia, Qian Xiao, Yuanzhen Mi, Shanshan Wei, Ziliang Wu, Bing Lin, Zhonglin Zhang

**Affiliations:** ^1^School of Clinical Medicine, Chengdu University of Traditional Chinese Medicine, Chengdu, China; ^2^Department of Oncology, Hospital of Chengdu University of Traditional Chinese Medicine, Chengdu, China; ^3^Health Management Center, Hospital of Chengdu University of Traditional Chinese Medicine, Chengdu, China; ^4^Department of Radiation Oncology, Radiation Oncology Key Laboratory of Sichuan Province, Sichuan Clinical Research Center for Cancer, Sichuan Cancer Hospital & Institute, Sichuan Cancer Center, Affiliated Cancer Hospital of University of Electronic Science and Technology of China, Chengdu, China

**Keywords:** randomized controlled trial, protocol, moxibustion, insomnia, breast cancer survivors

## Abstract

**Background:**

Insomnia (difficulty falling or staying asleep) is a common issue among breast cancer survivors, significantly impacting their quality of life. Current treatments, primarily pharmacological and psychological, have limitations: the former often causes side effects, while the latter faces accessibility barriers. Long-snake moxibustion (LSM), a traditional Chinese medicine (TCM) technique, involves applying moxibustion along the governor vessel, which is an important meridian in TCM that plays a key role in regulating brain function. LSM is characterized by its minimal side effects, ease of application, and cost-effectiveness, with preliminary studies supporting its potential for treating insomnia. This study aims to further investigate the therapeutic effectiveness of LSM in alleviating insomnia among breast cancer survivors and to explore its underlying mechanisms.

**Methods:**

This single-center, rater-masked, randomized controlled trial will enroll 100 breast cancer survivors with chronic insomnia, who will be randomly assigned in a 1:1 ratio to either the LSM group or a waitlist control group. During the 4-week treatment period, all participants will receive standard care, with the LSM group additionally receiving LSM treatment twice a week. The primary efficacy outcome is the change in Insomnia Severity Index (ISI) score at the end of the intervention. Secondary outcomes include changes in hypnotic medication use, Pittsburgh Sleep Quality Index (PSQI) scores, Piper Fatigue Scale (PFS) scores, and Functional Assessment of Cancer Therapy-Breast (FACT-B) scores. Mechanistic evaluations will assess serum biochemical markers, gut microbiota composition, and metabolomic profiles.

**Discussion:**

If proven effective, this trial will provide critical clinical evidence supporting LSM as a viable and accessible treatment for insomnia among breast cancer survivors. The findings could influence clinical practice by offering a non-pharmacological treatment option, improving patient outcomes, and reducing dependence on pharmacological interventions. Furthermore, exploring the underlying mechanisms may enhance our understanding of how LSM works, paving the way for future research.

**Clinical trial registration:**

http://itmctr.ccebtcm.org.cn/, identifier ITMCTR2024000578.

## 1 Introduction

Breast cancer represents a significant global health challenge for women, standing as the most commonly diagnosed malignancy and the leading cause of cancer-related mortality. Epidemiological data from 2020 revealed approximately 2.3 million new cases of breast cancer and 685,000 related deaths worldwide, highlighting the substantial burden of this disease (1). As a complex and heterogeneous condition, breast cancer manifests through various molecular subtypes, with hormone receptor-positive (HR+) breast cancer constituting the predominant molecular subtype, representing nearly 70% of all breast cancer cases (2). The pathogenesis of HR + breast cancer is intrinsically linked to estrogen signaling pathways, necessitating prolonged endocrine therapy typically spanning 5–10 years as a fundamental component of adjuvant treatment to reduce recurrence risk (3).

Among the spectrum of cancer survivorship issues, insomnia emerges as a particularly prevalent and clinically significant comorbidity, characterized by persistent difficulties in sleep initiation, maintenance, or non-restorative sleep despite adequate opportunity for sleep (4). This sleep disorder is strongly associated with multidimensional health consequences, including increased fatigue, cognitive impairment, immune dysregulation, and elevated risk of psychiatric comorbidities such as depression and anxiety disorders (5, 6). The critical role of sleep in maintaining physiological homeostasis is well-established, with studies suggesting that around 7 h of sleep per night is optima (7). Notably, among cancer survivors, the prevalence of insomnia is significantly higher in breast cancer patients, reaching approximately 62% (8), a phenomenon potentially mediated through neuroendocrine dysregulation secondary to endocrine therapeutic interventions (9).

Current therapeutic approaches for insomnia predominantly include pharmacological and psychological interventions (10). Pharmacological treatments often encompass benzodiazepines, non-benzodiazepine agents, barbiturates, and other sedatives (11). While these interventions demonstrate considerable short-term efficacy, their long-term application is generally discouraged due to potential risks of tolerance, dependency, and adverse side effects (12). Psychological interventions, particularly cognitive-behavioral therapy (CBT), have shown effectiveness in improving sleep quality among breast cancer patients (13). However, the substantial labor demands and elevated costs associated with CBT limit its accessibility (14), underscoring the necessity for alternative therapeutic strategies.

Moxibustion, a core component of traditional Chinese medicine (TCM), involves stimulating specific acupoints to treat various conditions and is recognized for its minimal side effects, operational simplicity, and cost-effectiveness. A systematic review has suggested that moxibustion may be effective in managing insomnia (15). Long-snake moxibustion (LSM), a specialized form of moxibustion applied along the governor vessel from GV14 (Dazhui) to DU2 (Yaoyu), is theorized in TCM to be associated with the brain and closely related to sleep regulation. Compared to conventional moxibustion, LSM offers broader acupoint stimulation with enhanced heat radiation and deeper tissue penetration (16). Additionally, existing studies have indicated the anti-tumor effects of moxibustion (17). Based on these considerations, we hypothesize that LSM could serve as a viable strategy for managing cancer-related insomnia.

To empirically investigate this hypothesis, we have developed a randomized controlled trial targeting breast cancer survivors, a population particularly susceptible to insomnia. This study will rigorously evaluate both the clinical efficacy and underlying mechanisms of LSM by analyzing changes in the Insomnia Severity Index (ISI), as well as examining serum biochemical markers, gut microbiota composition, and metabolomic profiles.

## 2 Methods

### 2.1 Study design

**Figure 1:**
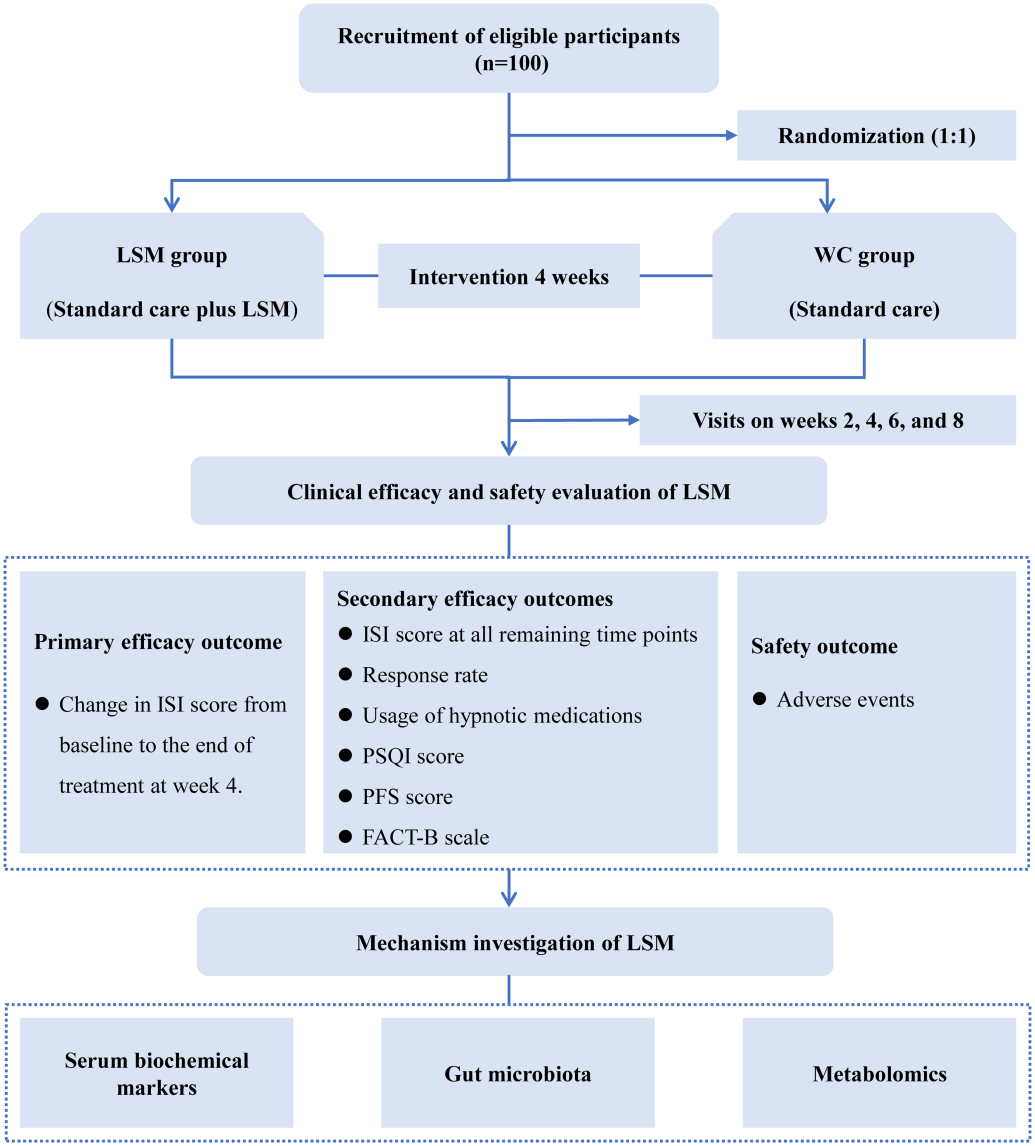
The flow diagram of this study. FACT-B, Functional Assessment of Cancer Therapy-Breast; ISI, Insomnia Severity Index; LSM, long-snake moxibustion; PFS, Piper Fatigue Scale; PSQI, Pittsburgh Sleep Quality Index; WC, waitlist control.

This study is designed as a single-center, rater-masked, parallel-arm, randomized controlled trial. We will recruit 100 breast cancer survivors diagnosed with insomnia from the Hospital of Chengdu University of Traditional Chinese Medicine. Participants will be randomly allocated in a 1:1 ratio to either the LSM intervention group or the waitlist control (WC) group. The study will span 9 weeks, comprising a 1-week baseline assessment phase, a 4-week intervention phase, and a 4-week post-treatment follow-up phase. Throughout the 4-week treatment period, both groups will receive standard care, with the LSM group additionally undergoing LSM therapy twice per week. This trial has been rigorously designed according to the Consolidated Standards of Reporting Trials (CONSORT) guidelines and will be reported following the 2013 Standard Protocol Items: Recommendations for Interventional Trials (SPIRIT) Checklist (see Supplementary material S1). The study flow is depicted in Figure 1.

### 2.2 Participants

#### 2.2.1 Recruitment

A comprehensive, multi-channel recruitment strategy will be implemented to enroll participants, utilizing posters, media advertisements, and clinical referrals. Prospective participants will undergo an initial screening through face-to-face interviews and baseline assessments conducted by the research team to confirm their eligibility for the study. In line with ethical standards, detailed information regarding the study’s objectives, intervention procedures, potential benefits, and associated risks will be provided to ensure participants give fully informed consent before enrollment.

#### 2.2.2 Inclusion criteria

1. Age range of 18 to 75 years.
2. Female patients with a pathological diagnosis of breast cancer, stages I to III, according to the American Joint Committee on Cancer (AJCC) 8th edition staging system.
3. Completion of primary breast cancer treatment (e.g., surgery, radiotherapy, chemotherapy, targeted therapy, or immunotherapy) for a minimum of 3 months, with the exception of ongoing endocrine therapy, which is permissible if administered for over 3 weeks.
4. No signs of cancer recurrence or new primary tumor development.
5. Diagnosis of chronic insomnia as defined by the Diagnostic and Statistical Manual of Mental Disorders, 5th edition (DSM-5) (18), according to criteria set by the American Psychiatric Association.
6. Insomnia severity ranging from mild to severe, represented by an ISI score of 8 or higher.

#### 2.2.3 Exclusion criteria

1. Diagnosis of other sleep disorders, such as sleep apnea, parasomnia, or narcolepsy.
2. Engagement in shift work or having irregular sleep-wake patterns.
3. Abnormal liver, kidney, or coagulation function tests, or the presence of serious diseases that may compromise the safety of the trial, including but not limited to respiratory disorders (e.g., severe asthma), cardiovascular diseases (e.g., coronary artery disease), and hematologic disorders (e.g., leukemia).
4. Pregnant or breastfeeding women.

### 2.3 Interventions

All enrolled participants will receive standardized care, comprising sleep hygiene education and, where applicable, hypnotic medication interventions. The sleep hygiene education will emphasize adherence to a consistent sleep schedule, optimizing the sleep environment, and avoiding the consumption of alcohol, nicotine, and caffeine for at least several hours before bedtime. Additionally, participants will be encouraged to engage in 180–300 min of low-to-moderate intensity physical activity per week, while avoiding exercise within 3 h of their intended sleep time. Emotional self-regulation strategies will be incorporated to further support sleep hygiene practices. Hypnotic medications, which are optional and not required for all patients, will be prescribed based on individual therapeutic needs and clinical assessments. These medications may include psychotropic drugs such as benzodiazepines, non-benzodiazepine agents, and narcotics. The type, dosage, and frequency of all prescribed medications will be meticulously documented to ensure comprehensive data collection.

In addition to the aforementioned standard care, participants in the LSM group will receive supplemental LSM therapy, administered twice weekly for a total of eight sessions. The LSM procedure will be conducted following standardized protocols established in previous clinical studies (19). The treatment area will extend along the spine from GV14 (Dazhui), located at the lower border of the spinous process of the seventh cervical vertebra, to DU2 (Yaoshu), located at the lower border of the spinous process of the second sacral vertebra, with a 3 cm extension on both sides of the spine. The acupuncture points within this 3 cm range include the 13 governor vessel points as well as 24 bladder meridian points. Detailed information on the specific acupuncture points is provided in Supplementary material S2. The LSM procedure will commence with the patient lying in a prone position, with the back fully exposed, followed by thorough disinfection of the targeted area. A 12 cm-wide, 70 cm-long piece of mulberry bark paper will then be placed along the spine’s midline, after which a trapezoidal ginger paste (base width: 6 cm, top width: 5 cm, height: 3 cm) will be evenly mounded from GV14 to DU2. A cylindrical moxa cone (5 cm in diameter and 3 cm in height) will subsequently be positioned on the ginger paste, aligned along its full length. The moxa cone will be ignited and allowed to burn completely before being replaced once more, ensuring sufficient heat stimulation across the treatment area. Each LSM session is estimated to last approximately 1.5 h.

### 2.4 Outcomes

#### 2.4.1 Primary efficacy outcome

The primary efficacy outcome will be the change in ISI score from baseline to the end of the 4-week treatment period. The ISI is a validated 7-item self-report measure that has demonstrated strong internal consistency and construct validity for evaluating insomnia severity in cancer patients (20). ISI scores are categorized as follows: 0–7 (no clinically significant insomnia), 8–14 (mild insomnia), 15–21 (moderate insomnia), and 22–28 (severe insomnia) (21). ISI assessments will be conducted at baseline, as well as at weeks 2, 4, 6, and 8 following the start of treatment.

#### 2.4.2 Secondary efficacy outcomes

1. Changes in ISI scores from baseline at all additional assessment time points.
2. Treatment response rate, defined as the percentage of participants achieving a reduction of ≥8 points in ISI score from baseline at each assessment (22).
3. Changes in hypnotic medication usage. To maintain consistency in subsequent analyses and reporting, all hypnotic doses will be converted to standardized diazepam equivalent doses. For participants using hypnotic medications, the average dosage over the preceding 2 weeks will be recorded at baseline, as well as at weeks 2, 4, 6, and 8 after treatment initiation.
4. Changes in Pittsburgh Sleep Quality Index (PSQI) scores. The PSQI is a widely used questionnaire to assess sleep quality over the past month, comprising seven components that evaluate specific sleep-related features (23). Scores range from 0 to 21, with 0–5 indicating good sleep quality, 6–10 suggesting moderate sleep quality, 11–15 reflecting poor sleep quality, and 16–21 indicating very poor sleep quality. Assessments will be conducted at baseline, as well as at weeks 4 and 8 post-treatment initiation.
5. Changes in Piper Fatigue Scale (PFS) scores. The PFS is a validated tool widely used to assess fatigue in cancer populations (24), evaluating four dimensions: behavioral, affective, sensory, and cognitive. Scores range from 0 to 10, with 0–3 indicating mild fatigue, 4–6 indicating moderate fatigue, and 7–10 indicating severe fatigue. Assessments will be conducted at baseline and at weeks 4 and 8 post-treatment initiation.
6. Quality of life, assessed using the Functional Assessment of Cancer Therapy-Breast (FACT-B) scale, which includes a general cancer subscale (27 items) and a breast cancer-specific subscale (9 items) (25). Higher scores on the FACT-B indicate better quality of life. Assessments will be conducted at baseline, and at weeks 4 and 8 post-treatment initiation.

#### 2.4.3 Mechanism outcomes

Blood and fecal samples will be collected from all participants at baseline and at the end of the treatment period (week 4). Fasting blood samples (20 mL) will be drawn from the antecubital vein between 7:00 and 9:00 AM. The samples will then be centrifuged at 3,000 rpm for 15 min at 4°C, and the resulting serum will be stored at −80°C. Fecal samples will be collected immediately after morning defecation, placed into sterilized fecal containers, and stored at −80°C. The mechanisms of LSM will be examined through serum biochemical markers, gut microbiota composition, and metabolomic profiling.

Serum biochemical markers will include neurotransmitters, inflammatory cytokines, and endocrine hormones. Neurotransmitters to be measured are 5-hydroxytryptamine (5-HT) and gamma-aminobutyric acid (GABA). Inflammatory markers will cover interleukin-1β (IL-1β), interleukin-6 (IL-6), and tumor necrosis factor-α (TNF-α). Hormonal analysis will focus on melatonin and hormones associated with the hypothalamic-pituitary-adrenal (HPA) axis, such as adrenocorticotropic hormone (ACTH), corticotropin-releasing hormone (CRH), and cortisol (CORT). All measurements will be conducted using enzyme-linked immunosorbent assays (ELISA).

Gut microbiota composition in fecal samples will be analyzed through 16S rRNA gene sequencing. α diversity will be assessed using observed ASV, Shannon, Simpson, and Chao1 indices, while β diversity will be evaluated through principal coordinates analysis (PCoA) based on the Bray–Curtis distance matrix. The linear discriminant analysis effect size (LEfSe) model will be applied to identify differentially abundant taxa between groups.

Metabolomic profiling of serum samples will be performed using non-targeted liquid chromatography-mass spectrometry (LC-MS) (26). Principal component analysis (PCA) and partial least squares discriminant analysis (PLS-DA) will be used to visualize metabolic patterns across groups. Key differential metabolites will be identified based on variable importance in projection (VIP) scores from the PLS-DA model. Additionally, non-targeted quantification of short-chain fatty acids (SCFAs) in fecal samples will be conducted via gas chromatography-mass spectrometry (GC-MS) (27).

#### 2.4.4 Safety outcome

All adverse events (AEs) occurring during the study will be comprehensively documented in the clinical research forms (CRFs), detailing the timing, symptoms, severity, interventions, prognosis, and any other relevant information. The severity of AEs will be assessed using the Common Terminology Criteria for Adverse Events (CTCAE) version 5.0 (28), as defined by the National Institutes of Health (NIH), with grades 3–5 classified as severe. The acupuncturist and study team will evaluate the potential causal relationship between the AEs and the LSM treatment. AEs potentially related to LSM may include, but are not limited to, blisters, erythema, pruritus, burns, and respiratory symptoms. Safety monitoring will involve regular assessments of the treatment site, temperature measurements to minimize the risk of burns and excessive erythema. Appropriate interventions will be implemented for all AEs, regardless of their association with LSM. In the event of severe AEs, urgent medical attention will be provided, the study intervention will be discontinued, and the research ethics committee will be notified within 24 h with a detailed report outlining the nature of the event, actions taken, and outcomes. The participant will be closely monitored until the serious AEs are adequately managed. The study team will conduct a thorough review of the severe AEs to determine whether modifications to the study protocol are necessary to prevent recurrence.

### 2.5 Participant timeline

**Figure 2:**
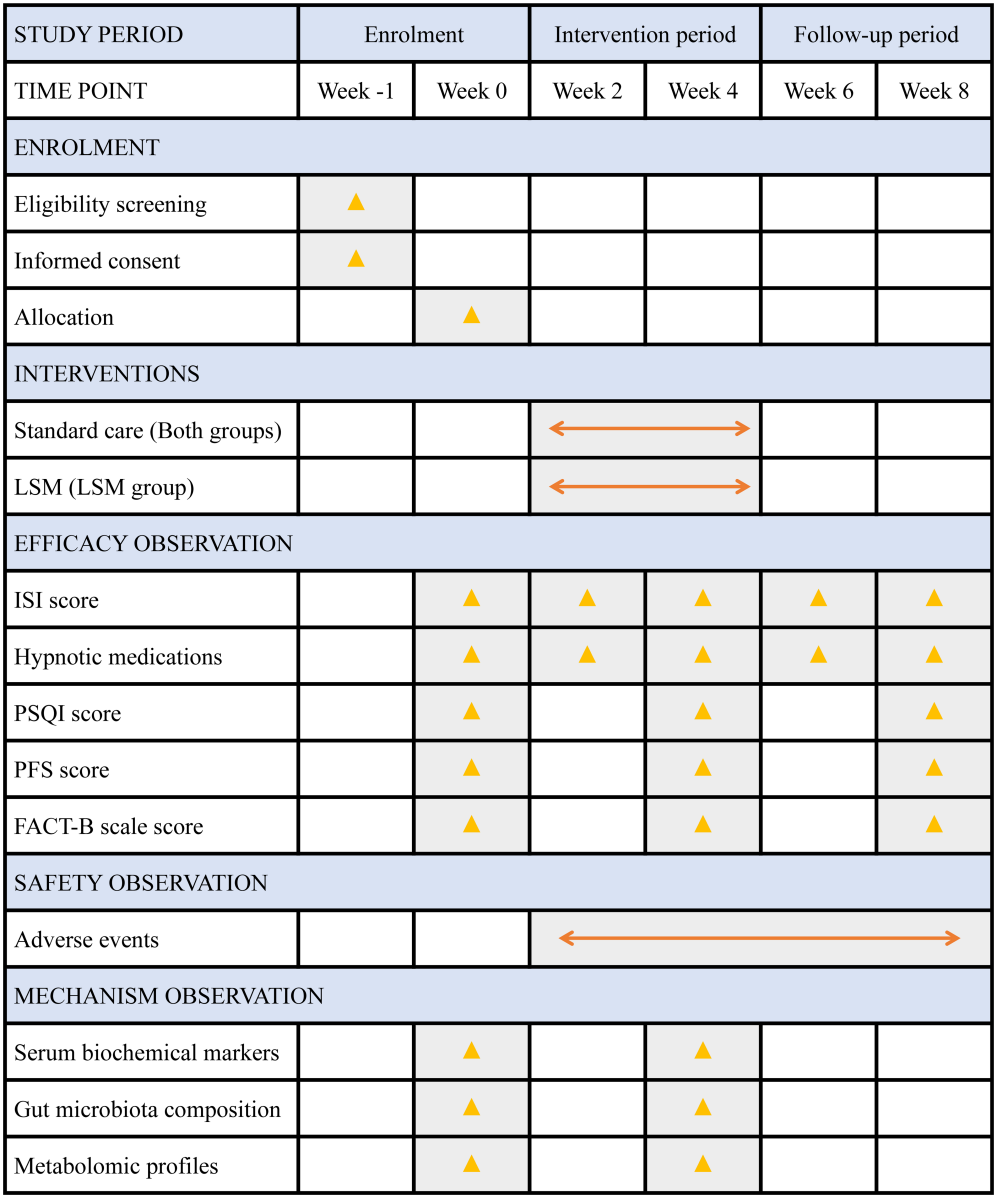
Detailed timeline of enrolment, interventions and assessments. FACT-B, Functional Assessment of Cancer Therapy-Breast; ISI, Insomnia Severity Index; LSM, long-snake moxibustion; PSQI, Pittsburgh Sleep Quality Index; PFS, Piper Fatigue Scale.

Figure 2 provides a detailed timeline outlining the schedule for participant enrollment, intervention procedures, outcome assessments, and systematic data collection throughout the study period.

### 2.6 Randomization

An independent biostatistician will generate the randomization sequence using a stratified block randomization design. Block sizes of 2 or 4 will be used, with stratification based on whether patients are currently receiving endocrine therapy (yes or no). Participants will be assigned to either the LSM or WC groups in a 1:1 ratio. The randomization codes will be sealed in sequentially numbered, opaque envelopes, which will be securely maintained by independent administrative staff. Participants will receive their envelopes in the order of enrollment to determine their group allocation.

### 2.7 Blinding

Given the distinct nature of LSM therapy, creating a placebo moxibustion is challenging. Consequently, this study will be conducted as an open-label trial, with both participants and acupuncturists aware of their treatment assignments. To mitigate potential bias, blinding will be maintained for outcome assessors, data managers, and statisticians. During assessments, outcome assessors will use CRFs containing participant names, while both participants and assessors will be instructed to avoid discussing treatment allocation. Once assessments are complete, independent administrative staff overseeing randomization will replace participant names with coded labels, and group assignments will be replaced with anonymized labels. The anonymized dataset will then be provided to data managers and statisticians for analysis. Since statisticians will only have access to the anonymized dataset, they will conduct the analysis without knowledge of treatment group assignments, ensuring unbiased interpretation of results. After data analysis is finalized, the identities associated with the codes will be disclosed to the research team for accurate interpretation of the results.

### 2.8 Sample size

To obtain a preliminary estimate of LSM’s efficacy, a pilot study was conducted with 12 participants assigned to each of the two groups, based on the rules of thumb (29). After the 4-week treatment period, ISI scores decreased by 6.82 ± 4.06 points in the LSM group and 3.24 ± 2.38 points in the WC group from baseline. The observed effect size (Cohen’s *d*) was 1.08, which, according to Cohen’s criteria (*d* = 0.2 as small, *d* = 0.5 as medium, and *d* = 0.8 as large), indicates a large effect. Based on these results, the sample size was calculated using the “Two-sample *t*-tests allowing unequal variance” module in PASS software, version 15.0, with a two-tailed alpha of 0.05 and a power of 0.90, while accounting for a 10% expected dropout rate. Previous studies have shown that endocrine therapy is an important risk factor for insomnia in breast cancer patients (9). Therefore, to account for potential heterogeneity in treatment response, the sample size was stratified by endocrine therapy status (yes vs. no). To ensure sufficient statistical power within both strata, the sample size was adjusted to 46 participants per group. Ultimately, considering the importance of using whole numbers for clear communication of results, and to further enhance the statistical power of the study, the sample size per group was rounded up to 50 participants.

### 2.9 Statistical analysis

The analysis will be conducted based on the intent-to-treat (ITT) population, defined as participants who complete baseline assessments and have at least one follow-up measurement. The Shapiro–Wilk test will be employed to assess the normality of the data distribution. For continuously measured repeated data, such as ISI scores, PSQI scores, PFS scores, FACT-B scores, and hypnotic medication dosages, a mixed-effects model adjusted for baseline values will be used to compare longitudinal changes from baseline across follow-up time points between groups. The model will include fixed effects for endocrine therapy status (yes or no), time, group, and the interaction between time and group, while treating individual participants as random effects. Given that mixed-effects models can accommodate participants with at least one outcome measurement, no imputation will be performed for missing values in the primary analysis (30).

For non-repeated continuous variables, such as serum biochemical markers and gut microbiota abundance, between-group comparisons will be performed using either the *t*-test or Mann–Whitney *U* test, depending on data distribution. Categorical variables, including treatment response rates and incidence of adverse events, will be analyzed using chi-square or Fisher’s exact tests. Correlation analyses between ISI scores, differential serum biochemical markers, differential metabolites, and key gut microbiota will be performed using Pearson or Spearman correlation coefficients. Missing values for data analyzed using non-mixed effect models will be managed using multiple imputation methods.

For the primary efficacy outcome (the change in the ISI score from baseline to the end of the 4-week treatment period), subgroup analyses will be conducted based on hypnotic medication use (yes, no) and insomnia severity (mild, moderate, severe) to further explore potential variations in the treatment effects of LSM across different subgroups. Additionally, sensitivity analyses will be performed for the primary efficacy outcome. Specifically, if missing values or non-normally distributed data are present, multiple imputation methods will be used to assess the impact of missing data, and generalized estimating equations will be employed as a non-parametric alternative for non-normally distributed data. Pre-specified per-protocol analyses will also be conducted. The per-protocol set will include all participants who completed the intervention and follow-up according to the study protocol. The results of the primary analysis will be compared with those from these sensitivity analyses to assess robustness.

All statistical analyses will be carried out using SPSS (version 26.0) and R (version 4.3.1). Two-tailed significance testing will be applied, with a *p*-value threshold of ≤ 0.05 indicating statistical significance.

### 2.10 Quality control and data monitoring

Before participant enrollment, all study personnel will undergo comprehensive training sessions aimed at ensuring standardized implementation and enhancing the reliability of study outcomes. These sessions will cover key aspects such as study design, eligibility criteria, intervention procedures, outcome measurement, and accurate data documentation in CRFs.

The LSM procedures will be performed by acupuncturists with a minimum of 3 years of moxibustion experience. To minimize inter-practitioner variability, all practitioners will undergo a standardized training program focusing on the detailed application of the LSM procedure, including ginger paste preparation, moxa cone positioning, mulberry bark paper placement, and the treatment area covered along the spine. Practitioners will be required to demonstrate competency in following the treatment protocol through supervised practice sessions before performing the treatment on study participants. To further ensure treatment fidelity, a senior clinician will monitor and audit the LSM procedures periodically throughout the study. Any deviations from the standardized protocol will be addressed promptly to maintain consistency across practitioners and sessions.

Data collection will be conducted using paper CRFs to systematically document study-related information. All paper records will be securely stored in locked cabinets under the supervision of authorized personnel. Electronic data entry will be managed through a double-entry system, conducted by two independent and experienced staff members. Data will be stored in an encrypted research database, accessible only to authorized research personnel. Strict measures will be implemented to ensure data confidentiality and prevent unauthorized access or breaches. The Chengdu University of Traditional Chinese Medicine Research Ethics Committee will perform regular audits to verify data accuracy, monitor trial conduct, assess study progress, and ensure compliance with ethical standards and protocol requirements.

## 3 Discussion

This randomized controlled trial aims to evaluate both the clinical efficacy and the potential mechanisms of LSM therapy in treating insomnia among breast cancer survivors. A key feature of this study is the use of a multi-omics approach, which will analyze changes in serum biochemical markers, gut microbiota composition, and metabolomic profiles to provide substantial theoretical support for the therapeutic effects of LSM.

Insomnia is associated with dysregulation across several physiological systems, particularly the nervous, immune, and endocrine systems. This study will focus on key serum biochemical markers, including neurotransmitters (5-HT and GABA), inflammatory cytokines (IL-1β, IL-6, TNF-α), and endocrine hormones (melatonin and hormones related to the HPA axis), as these have been implicated in the pathophysiology of insomnia. Specifically, 5-HT and GABA act as critical inhibitory neurotransmitters that help modulate excessive neuronal activity, and their levels are often reduced in individuals with insomnia (31, 32). Retrospective clinical research has suggested that moxibustion may improve insomnia in coronary heart disease patients by increasing serum levels of 5-HT and GABA (33). Regarding the link between inflammatory cytokines and sleep disturbances, IL-1β, IL-6, and TNF-α are commonly studied markers. A literature review underscores the strong association between elevated levels of these cytokines and the worsening of insomnia symptoms (34). Research has shown that IL-1β and TNF-α may exacerbate sleep disturbances by inhibiting the synthesis and release of neurotransmitters GABA and 5-HT (35). Previous studies have consistently demonstrated that moxibustion may exert anti-inflammatory effects, potentially reducing these cytokines (19, 36).

Melatonin, a neurohormone produced by the pineal gland, plays a critical role in regulating circadian rhythms and the sleep-wake cycle (37). Reduced melatonin levels are frequently associated with insomnia (38). Observational studies suggest that moxibustion may increase serum melatonin levels, potentially alleviating symptoms related to aging (39). The HPA axis, a key component of the neuroendocrine system, is activated in response to stress (40). Hyperactivity of the HPA axis can compromise sleep quality by increasing fragmentation, reducing slow-wave sleep, and shortening total sleep duration (41). Elevated CORT levels are often considered a “gold standard” marker of HPA axis activation (42), have been shown to directly suppress melatonin secretion (43), thereby disrupting circadian rhythms and exacerbating sleep disturbances. Previous studies have shown that moxibustion can reduce serum levels of ACTH, CORT, and CRH in animal models, suggesting that it may improve insomnia by rebalancing HPA axis function (44). Collectively, these findings support the hypothesis that LSM may alleviate insomnia through its regulatory effects on neurotransmitters, inflammatory cytokines, and endocrine hormones.

Emerging evidence indicates that gut microbiota may influence sleep-wake cycles through immune modulation, hormonal signaling, bacterial metabolites, and neural communication pathways (45). Significant alterations in the diversity, composition, and structure of the gut microbiota have been observed in individuals with insomnia. For instance, a recent Mendelian randomization study identified that the microbial taxa *Enterorhabdus* and *Paraprevotella* were associated with an increased risk of insomnia, whereas *Coprobacter*, *Desulfovibrio*, *Flavonifractor*, *Olsenella*, *Odoribacter*, and *Oscillibacter* were linked to a decreased risk (46). One clinical study reported that insomnia patients, compared to healthy controls, exhibited reduced α-and β-diversity, increased *Bacteroidetes*, and a decreased *Firmicutes*/*Bacteroidetes* ratio (47). Recent studies further suggest that *Bacteroidetes* dominate the gut microbiota of insomnia patients, and good sleep is closely associated with lower *Bacteroidetes* abundance (48). Another study found that the relative abundances of *Lactobacillus*, *Streptococcus*, and *L. crispatus* were significantly higher in insomnia patients compared to non-insomniacs (49). An animal study provided preliminary evidence that moxibustion may restore the normal *Firmicutes*/*Bacteroidetes* ratio and reduce the abundance of *Lactobacillus* and *Proteobacteria*, thereby alleviating chronic fatigue syndrome in rats (50).

Metabolic processes are closely linked to insomnia. A clinical study using serum metabolomics analysis in insomnia patients revealed significant disruptions in five metabolic pathways: glutathione metabolism; alanine, aspartate, and glutamate metabolism; aminoacyl-tRNA biosynthesis; nitrogen metabolism; and glycerophospholipid metabolism (49). Another clinical trial found that later sleep timings were associated with increased levels of various metabolites involved in amino acid metabolism, including branched-chain amino acids and their gamma-glutamyl derivatives (51). SCFAs, the primary bioactive metabolites produced by the gut microbiota, mainly include acetate, propionate, and butyrate (52). Growing evidence suggests that reduced SCFA concentrations are associated with insomnia (53, 54). In animal studies, moxibustion has been shown to promote the growth of beneficial gut probiotics and elevate SCFA levels, potentially delaying aging in Wistar rats (55). These findings collectively support the hypothesis that LSM may alleviate insomnia through its modulatory effects on both gut microbiota composition and metabolic pathways.

Furthermore, research indicates a strong association between several systemic diseases and an increased risk of insomnia, including hypertension (56), depression (57), and anxiety (58). Meta-analyses suggest that moxibustion may positively affect blood pressure (59) and improve symptoms of depression and anxiety in cancer patients (60). Therefore, LSM may alleviate insomnia symptoms by modulating these associated diseases.

In summary, this study employs a multidisciplinary approach to explore the potential effects of LSM on biological systems associated with insomnia in breast cancer survivors, aiming to build a strong evidence base. The findings are expected to provide valuable insights for clinical practice and validate the potential of LSM as an effective non-pharmacological treatment for insomnia. By addressing challenges related to the significant side effects of pharmacotherapy and the limited accessibility of cognitive behavioral therapy, LSM may offer a more effective and accessible alternative. However, several limitations must be acknowledged. First, as a single-center trial, the generalizability of the findings may be limited, and future multi-center studies will help confirm these results in a more diverse patient population. Second, due to the current lack of a well-established LSM placebo tool, this study adopted an open-label design. The absence of blinding may introduce bias, particularly in subjective outcome measures, where participants’ expectations could influence their reported outcomes, potentially compromising the accuracy of the results. Future studies should focus on developing an appropriate placebo tool for LSM and employ a double-blind trial design to more objectively assess its efficacy. Lastly, the relatively short follow-up period of 4 weeks limits our ability to evaluate the long-term effects and sustainability of the treatment. Longer follow-up periods are recommended in future research to assess the durability of the treatment benefits.
