# Supplementary material for: Efficacy and mechanism of long-snake moxibustion for treating insomnia in breast cancer survivors: study protocol for a randomized controlled trial
Source: Front Neurol. 2025 Apr 30;16:1524412. doi: 10.3389/fneur.2025.1524412 (PMC12074938; doi:10.3389/fneur.2025.1524412)
Supplement: Supplementary file 1 [file Data_Sheet_1.pdf]

SPIRIT Checklist: Recommended Items to Address in a Clinical Trial Protocol and Related Documents

Section/Item<br>Administrative information	Item Number	Description
Title	1	Descriptive title identifying the study design, population, interventions, and, if applicable, trial acronym<br>Efficacy and Mechanism of Long-Snake Moxibustion for Treating Insomnia in Breast Cancer Survivors: Study Protocol for a Randomized Controlled Trial
Trial registration	2a	Trial identifier and registry name. If not yet registered, name of intended registry.<br>http://itmctr.ccebtcm.org.cn/, identifier ITMCTR2024000578
	2b	All items from the World Health Organization Trial Registration Data Set (Appendix Table, available at www.annals.org)<br>Not applicable for this trial.
Protocol version	3	Date and version identifier<br>Date August 1, 2024 and version 3.0
Funding	4	Sources and types of financial, material, and other support<br>This research is funded by the 2023 Science and Technology Development Fund of the Hospital of Chengdu University of Traditional Chinese Medicine (Grant Nos. 23TS17 and 23HL24) and the 2024 Sichuan Provincial

		Cadre Healthcare Research Project (Grant No. 2024-501).
Roles and responsibilities	5a	Names, affiliations, and roles of protocol contributors<br><br>Cuicui Gong 1, Huakang Li 1, Qi Xiao 2, Pengxuan Gu 1, yunjing jia 1, Qian Xiao 1, Yuanzhen Mi 1, Shanshan Wei 3, Ziliang Wu 2, Bing Lin 2, Zhonglin Zhang 3<br><br>1. School of Clinical Medicine, Chengdu University of Traditional Chinese Medicine, Chengdu, Sichuan Province, China.<br><br>2. Health Management Center, Hospital of Chengdu University of Traditional Chinese Medicine, Chengdu, Sichuan Province, China.<br><br>3. Department of Radiation Oncology, Radiation Oncology Key Laboratory of Sichuan Province, Sichuan Clinical Research Center for Cancer, Sichuan Cancer Hospital & Institute, Sichuan Cancer Center, Affiliated Cancer Hospital of University of Electronic Science and Technology of China, Chengdu, Sichuan Province, China.<br><br>In this research, Cuicui Gong contributed to conceptualization, formal analysis, methodology, and the writing of the original draft, as well as review and editing. Qi Xiao was responsible for data curation, funding acquisition, project administration, resources, and review and editing of the manuscript. Huakang Li contributed to conceptualization, methodology, software development, drafting the original manuscript, and review and editing. Pengxuan Gu participated in investigation, methodology, and review and editing of the writing. Yunjing Jia

		contributed to formal analysis, investigation, and review and editing of the manuscript. Qian Xiao was involved in methodology, software, visualization, and review and editing. Bing Lin supported conceptualization, funding acquisition, project administration, resources, supervision, and review and editing. Yuanzhen Mi contributed to investigation, methodology, and review and editing, while Shanshan Wei provided resources and engaged in review and editing. Zhonglin Zhang contributed to conceptualization, project administration, resources, supervision, and review and editing, and Ziliang Wu participated in funding acquisition, project administration, supervision, and review and editing.
	5b	Name and contact information for the trial sponsor<br>Science and Technology Development Fund of the Hospital of Chengdu University of Traditional Chinese Medicine, No. 39, Shi-er-qiao Road, Chengdu 610072, Sichuan Province, China. Phone: +86 28-87783242.<br>Sichuan Provincial Cadre Health Care Committee Office, No. 39, Shangwangjiaguai Street, Chengdu 610041, Sichuan Province, China. Phone: +86 28-86131488.
	5c	Role of study sponsor and funders, if any, in study design; collection, management, analysis, and interpretation of data; writing of the report; and the decision to submit the report for publication, including whether they will have ultimate authority over any of these activities<br>Funders of this study have no role in any abovementioned activities.
	5d	Composition, roles, and responsibilities of the coordinating center, steering committee, end point adjudication

		committee, data management team, and other individuals or groups overseeing the trial, if applicable (see item 21a for DMC)<br>The Chengdu University of Traditional Chinese Medicine Research Ethics Committee will perform regular audits to verify data accuracy.
**Introduction**		
Background and rationale	6a	Description of research question and justification for undertaking the trial, including summary of relevant studies (published and unpublished) examining benefits and harms for each intervention<br>Insomnia is a common issue among breast cancer survivors, significantly impacting their quality of life. Current treatments, primarily pharmacological and psychological, have limitations: the former often causes side effects, while the latter faces accessibility barriers. Long-Snake Moxibustion (LSM), a traditional Chinese medicine modality, has shown promise as a non-pharmacological approach for insomnia, characterized by its minimal side effects, simplicity, and cost-effectiveness. This study aims to evaluate the efficacy and investigate the mechanisms of LSM in alleviating insomnia among breast cancer survivors.
	6b	Explanation for choice of comparators<br>Standard care as the comparators in this study.
Objectives	7	Specific objectives or hypotheses<br>The primary objective of this study is to evaluate the efficacy of Long-Snake Moxibustion (LSM) in reducing

		insomnia severity among breast cancer survivors, as reflected by changes in the Insomnia Severity Index (ISI) score at the intervention's conclusion. Secondary objectives include changes in hypnotic medication use, Pittsburgh Sleep Quality Index (PSQI) scores, Piper Fatigue Scale (PFS) scores, and Functional Assessment of Cancer Therapy-Breast (FACT-B) scores. Additionally, mechanistic objectives aim to explore potential mechanisms by which LSM alleviates insomnia, including analysis of changes in serum biochemical markers, shifts in gut microbiota composition, and alterations in metabolomic profiles associated with LSM treatment. The hypotheses are that LSM will significantly reduce insomnia severity compared to standard care alone, lead to lower hypnotic medication use and improved quality of life, and produce measurable changes in serum biochemical markers, gut microbiota, and metabolomic profiles, indicating biological mechanisms underlying its effects on insomnia.
Trial design	8	Description of trial design, including type of trial (e.g., parallel group, crossover, factorial, single group), allocation ratio, and framework (e.g., superiority, equivalence, noninferiority, exploratory)<br>This study is designed as a single-center, rater-masked, parallel-arm, randomized controlled trial.
**Methods**		
Participants, interventions, and outcomes		
Study setting	9	Description of study settings (e.g., community clinic, academic hospital) and list of countries where data will be collected. Reference to where list of study sites can be obtained

		A total of 100 breast cancer survivors with a diagnosis of insomnia will be recruited from the Hospital of Chengdu University of Traditional Chinese Medicine.
Eligibility criteria	10	Inclusion and exclusion criteria for participants. If applicable, eligibility criteria for study centers and individuals who will perform the interventions (e.g., surgeons, psychotherapists)<br>**Inclusion criteria**<br>(1). Age range of 18 to 75 years;<br>(2). Female patients with a pathological diagnosis of breast cancer, stages I to III, according to the American Joint Committee on Cancer (AJCC) 8th edition staging system;<br>(3). Completion of primary breast cancer treatment (e.g., surgery, radiotherapy, chemotherapy, targeted therapy, or immunotherapy) for a minimum of 3 months, with the exception of ongoing endocrine therapy, which is permissible if administered for over 3 weeks;<br>(4). No signs of cancer recurrence or new primary tumor development;<br>(5). Diagnosis of chronic insomnia as defined by the Diagnostic and Statistical Manual of Mental Disorders, Fifth Edition (DSM-5) (18), according to criteria set by the American Psychiatric Association;<br>(6). Insomnia severity ranging from mild to severe, represented by an ISI score of 8 or higher.<br>**Exclusion criteria**<br>(1). Diagnosis of other sleep disorders, such as sleep apnea, parasomnia, or narcolepsy;

		(2). Engagement in shift work or having irregular sleep-wake patterns;<br>(3). Abnormal liver, kidney, or coagulation function tests, or the presence of serious diseases that may compromise the safety of the trial, including but not limited to respiratory disorders (e.g., severe asthma), cardiovascular diseases (e.g., coronary artery disease), and hematologic disorders (e.g., leukemia);<br>(4). Pregnant or breastfeeding women.
Interventions	11a	Interventions for each group with sufficient detail to allow replication, including how and when they will be administered<br>All enrolled participants will receive standardized care, comprising sleep hygiene education and, where applicable, hypnotic medication interventions. The sleep hygiene education will emphasize adherence to a consistent sleep schedule, optimizing the sleep environment, and avoiding the consumption of alcohol, nicotine, and caffeine for at least several hours before bedtime. Additionally, participants will be encouraged to engage in 180–300 minutes of low-to-moderate intensity physical activity per week, while avoiding exercise within three hours of their intended sleep time. Emotional self-regulation strategies will be incorporated to further support sleep hygiene practices. Hypnotic medications, which are optional and not required for all patients, will be prescribed based on individual therapeutic needs and clinical assessments. These medications may include psychotropic drugs such as benzodiazepines, non-benzodiazepine agents, and narcotics. The type, dosage, and frequency of all prescribed medications will be meticulously documented to ensure comprehensive data

		collection.<br>In addition to the aforementioned standard care, participants in the LSM group will receive supplemental LSM therapy, administered twice weekly for a total of eight sessions. The LSM procedure will be conducted following standardized protocols established in previous clinical studies. The treatment area will extend along the spine from GV14 (Dazhui), located at the lower border of the spinous process of the seventh cervical vertebra, to DU2 (Yaoshu), located at the lower border of the spinous process of the second sacral vertebra, with a 3 cm extension on both sides of the spine. The acupuncture points within this 3 cm range include the 13 governor vessel points as well as 24 bladder meridian points. Detailed information on the specific acupuncture points is provided in Supplementary Material S2. The LSM procedure will commence with the patient lying in a prone position, with the back fully exposed, followed by thorough disinfection of the targeted area. A 12 cm-wide, 70 cm-long piece of mulberry bark paper will then be placed along the spine's midline, after which a trapezoidal ginger paste (base width: 6 cm, top width: 5 cm, height: 3 cm) will be evenly mounded from GV14 to DU2. A cylindrical moxa cone (5 cm in diameter and 3 cm in height) will subsequently be positioned on the ginger paste, aligned along its full length. The moxa cone will be ignited and allowed to burn completely before being replaced once more, ensuring sufficient heat stimulation across the treatment area. Each LSM session is estimated to last approximately 1.5 hours.
	11b	Criteria for discontinuing or modifying allocated interventions for a given trial participant (e.g., drug dose change

		in response to harms, participant request, or improving/worsening disease)<br>(1) Participants have the right to voluntarily withdraw from the trial at any stage for any reason;<br>(2) Participants who experience severe adverse events that preclude continuation in the trial;<br>(3) Death.
	11c	Strategies to improve adherence to intervention protocols, and any procedures for monitoring adherence (e.g., drug tablet return, laboratory tests)<br>To enhance adherence to intervention protocols and ensure effective monitoring, we will implement several key strategies: delivering clear communication and education to help participants understand the importance of protocol compliance, conducting regular follow-ups for ongoing support, and offering small incentives to encourage consistent participation. Together, these strategies are designed to improve adherence, minimize deviations, and ensure the reliability of study outcomes.
	11d	Relevant concomitant care and interventions that are permitted or prohibited during the trial<br>During the study, subjects will be prohibited from using any treatment methods other than those specified in the protocol for insomnia management (e.g., Traditional Chinese medicine preparations, cognitive-behavioral therapy, etc.).

Outcomes	12	Primary, secondary, and other outcomes, including the specific measurement variable (e.g., systolic blood pressure), analysis metric (e.g., change from baseline, final value, time to event), method of aggregation (e.g., median, proportion), and time point for each outcome. Explanation of the clinical relevance of chosen efficacy and harm outcomes is strongly recommended<br>The primary efficacy outcome is the change in Insomnia Severity Index (ISI) score at the end of the intervention. Secondary outcomes include changes in hypnotic medication use, Pittsburgh Sleep Quality Index (PSQI) scores, Piper Fatigue Scale (PFS) scores, and Functional Assessment of Cancer Therapy-Breast (FACT-B) scores. Mechanistic evaluations will assess serum biochemical markers, gut microbiota composition, and metabolomic profiles.
Participant timeline	13	Time schedule of enrollment, interventions (including any runins and washouts), assessments, and visits for participants. A schematic diagram is highly recommended (Figure).<br>As shown in figure 1 and figure 2.
Sample size	14	Estimated number of participants needed to achieve study objectives and how it was determined, including clinical and statistical assumptions supporting any sample size calculations<br>To obtain a preliminary estimate of LSM's efficacy, a pilot study was conducted with 12 participants assigned to each of the two groups, based on the rules of thumb. After the 4-week treatment period, ISI scores decreased by 6.82±4.06 points in the LSM group and 3.24±2.38 points in the WC group from baseline. The observed effect

		size (Cohen's d) was 1.08, which, according to Cohen's criteria (d = 0.2 as small, d = 0.5 as medium, and d = 0.8 as large), indicates a large effect. Based on these results, the sample size was calculated using the "Two-Sample T-Tests Allowing Unequal Variance" module in PASS software, version 15.0, with a two-tailed alpha of 0.05 and a power of 0.90, while accounting for a 10% expected dropout rate. Previous studies have shown that endocrine therapy is an important risk factor for insomnia in breast cancer patients . Therefore, to account for potential heterogeneity in treatment response, the sample size was stratified by endocrine therapy status (yes vs. no). To ensure sufficient statistical power within both strata, the sample size was adjusted to 46 participants per group. Ultimately, considering the importance of using whole numbers for clear communication of results, and to further enhance the statistical power of the study, the sample size per group was rounded up to 50 participants.
Recruitment	15	Strategies for achieving adequate participant enrollment to reach target sample size<br>A comprehensive, multi-channel recruitment strategy will be implemented to enroll participants, utilizing posters, media advertisements, and clinical referrals.
Assignment of interventions (for controlled trials)		
Allocation Sequence generation	16a	Method of generating the allocation sequence (e.g., computer-generated random numbers), and list of any factors for stratification. To reduce predictability of a random sequence, details of any planned restriction (e.g., blocking) should be provided in a separate document that is unavailable to those who enroll participants or assign interventions.

		An independent biostatistician will generate the randomization sequence using a stratified block randomization design. Block sizes of 2 or 4 will be used, with stratification based on whether patients are currently receiving endocrine therapy (yes or no). Participants will be assigned to either the long-snake moxibustion (LSM) or waitlist control (WC)groups in a 1:1 ratio.
Allocation concealment mechanism	16b	Mechanism of implementing the allocation sequence (e.g., central telephone; sequentially numbered, opaque, sealed envelopes), describing any steps to conceal the sequence until interventions are assigned<br>The randomization codes will be sealed in sequentially numbered, opaque envelopes, which will be securely maintained by independent administrative staff.
Implementation	16c	Who will generate the allocation sequence, who will enroll participants, and who will assign participants to interventions<br>An independent biostatistician, not involved in treatment or assessment, will generate the allocation sequence. Pengxuan Gu, Yunjing Jia, and Yuanzhen Mi will recruit participants, while an independent administrative staff member, responsible for safeguarding the randomization code, will allocate participants to interventions according to the pre-prepared allocation sequence.
Blinding (masking)	17a	Who will be blinded after assignment to interventions (e.g., trial participants, care providers, outcome assessors, data analysts), and how<br>This study will be conducted as an open-label trial, with both participants and acupuncturists aware of treatment

		assignments. To minimize potential bias, outcome assessors, data managers, and statisticians will remain blinded to treatment allocation.
	17b	If blinded, circumstances under which unblinding is permissible, and procedure for revealing a participant's allocated intervention during the trial<br>During assessments, outcome assessors will use clinical research forms (CRFs) containing participant names, while both participants and assessors will be instructed to avoid discussing treatment allocation. Once assessments are complete, independent administrative staff overseeing randomization will replace participant names with coded labels, and group assignments will be replaced with anonymized labels. The anonymized dataset will then be provided to data managers and statisticians for analysis. After data analysis is finalized, the identities associated with the codes will be disclosed to the research team for accurate interpretation of the results.
Data collection, management, and analysis		
Data collection methods	18a	Plans for assessment and collection of outcome, baseline, and other trial data, including any related processes to promote data quality (e.g., duplicate measurements, training of assessors) and a description of study instruments (e.g., questionnaires, laboratory tests) along with their reliability and validity, if known. Reference to where data collection forms can be found, if not in the protocol.<br>All personnel involved in the study implementation will receive standardized training, utilizing uniform recording methods and evaluation criteria. Comprehensive, detailed, and practical standard operating procedures

		will be developed and rigorously adhered to throughout the study. Investigators will accurately and thoroughly document all information in the case report forms (CRFs) in strict accordance with CRFs completion requirements, ensuring the authenticity and reliability of the data. All observations and findings will undergo thorough review to ensure data reliability, confirming that all clinical trial conclusions are drawn directly from the original data.
	18b	Plans to promote participant retention and complete follow-up, including list of any outcome data to be collected for participants who discontinue or deviate from intervention protocols<br>Plans to promote participant retention and ensure complete follow-up will include proactive strategies, such as regular communication, personalized reminders, and support resources.
Data management	19	Plans for data entry, coding, security, and storage, including any related processes to promote data quality (e.g., double data entry; range checks for data values). Reference to where details of data management procedures can be found, if not in the protocol.<br><br>Data collection will be conducted using paper case report forms (CRFs) to systematically document study-related information. All paper records will be securely stored in locked cabinets under the supervision of authorized personnel. Electronic data entry will be managed through a double-entry system, conducted by two independent and experienced staff members. Data will be stored in an encrypted research database, accessible only to authorized research personnel. Strict measures will be implemented to ensure data confidentiality and prevent unauthorized access or breaches. The Chengdu University of Traditional Chinese Medicine Research Ethics Committee will perform regular audits to verify data accuracy, monitor trial conduct, assess study progress, and ensure compliance with ethical standards and protocol requirements.

Statistical methods	20a	Statistical methods for analyzing primary and secondary outcomes. Reference to where other details of the statistical analysis plan can be found, if not in the protocol.<br><br>The analysis will be conducted based on the intent-to-treat (ITT) population, defined as participants who complete baseline assessments and have at least one follow-up measurement. For continuously measured repeated data, such as ISI scores, FACT-B scores, and hypnotic medication dosages, a mixed-effects model adjusted for baseline values will be used to compare longitudinal changes from baseline across follow-up time points between groups. The model will include fixed effects for endocrine therapy status (yes or no), time, group, and the interaction between time and group, while treating individual participants as random effects. Since mixed-effects models accommodate participants with at least one outcome measurement, no imputation will be performed for missing values .<br><br>For non-repeated continuous variables, such as serum biochemical markers and gut microbiota abundance, between-group comparisons will be performed using either the t-test or Mann-Whitney U test, depending on data distribution. Categorical variables, including treatment response rates and incidence of adverse events, will be analyzed using chi-square or Fisher's exact tests. Correlation analyses between ISI scores, differential serum biochemical markers, differential metabolites, and key gut microbiota will be performed using Pearson or Spearman correlation coefficients. Missing values for data analyzed using non-mixed effect models will be managed using multiple imputation methods.<br><br>All statistical analyses will be carried out using SPSS (version 26.0) and R (version 4.3.1). Two-tailed significance testing will be applied, with a P-value threshold of ≤ 0.05 indicating statistical significance.

	20b	Methods for any additional analyses (e.g., subgroup and adjusted analyses)<br>For the primary efficacy outcome (the change in the ISI score from baseline to the end of the 4-week treatment period), subgroup analyses will be conducted based on hypnotic medication use (yes, no) and insomnia severity (mild, moderate, severe) to further explore potential variations in the treatment effects of LSM across different subgroups. Additionally, sensitivity analyses will be performed for the primary efficacy outcome. Specifically, if missing values or non-normally distributed data are present, multiple imputation methods will be used to assess the impact of missing data, and generalized estimating equations will be employed as a non-parametric alternative for non-normally distributed data. Pre-specified per-protocol analyses will also be conducted. The per-protocol set will include all participants who completed the intervention and follow-up according to the study protocol. The results of the primary analysis will be compared with those from these sensitivity analyses to assess robustness.
	20c	Definition of analysis population relating to protocol nonadherence (e.g., as-randomized analysis), and any statistical methods to handle missing data (e.g., multiple imputation)<br>Missing data will be analyzed by multiple imputation.
Monitoring		
Data monitoring	21a	Composition of DMC; summary of its role and reporting structure; statement of whether it is independent from the sponsor and competing interests; and reference to where further details about its charter can be found, if not

		in the protocol. Alternatively, an explanation of why a DMC is not needed.<br>The Research Ethics Committee of the Hospital of Chengdu University of Traditional Chinese Medicine will conduct regular audits to verify data accuracy, monitor trial conduct, assess study progress, and ensure adherence to ethical standards and protocol requirements. These audits are conducted independently of the sponsors and research team, with no competing interests involved.
	21b	Description of any interim analyses and stopping guidelines, including who will have access to these interim results and make the final decision to terminate the trial.<br>The Research Ethics Committee of the Hospital of Chengdu University of Traditional Chinese Medicine will oversee the treatment process and ensure the integrity of the trial data, conducting interim analyses to verify compliance with protocol principles. Comprising six renowned experts from various fields, the committee monitors trial performance and safety every six months. It will have the right to access interim results and holds the final authority to determine whether the trial should be terminated.
Harms	22	Plans for collecting, assessing, reporting, and managing solicited and spontaneously reported adverse events and other unintended effects of trial interventions or trial conduct<br>All adverse events (AEs) occurring during the study will be comprehensively documented in the clinical research forms (CRFs), detailing the timing, symptoms, severity, interventions, prognosis, and any other relevant information. The severity of AEs will be assessed using the Common Terminology Criteria for Adverse Events (CTCAE) version 5.0, as defined by the National Institutes of Health (NIH), with grades 3–5 classified as severe. The acupuncturist and study team will evaluate the potential causal relationship between the AEs and the LSM treatment. AEs potentially related to LSM may include, but are not limited to, blisters, erythema, pruritus, burns,

		and respiratory symptoms. Safety monitoring will involve regular assessments of the treatment site, temperature measurements to minimize the risk of burns and excessive erythema. Appropriate interventions will be implemented for all AEs, regardless of their association with LSM. In the event of severe AEs, urgent medical attention will be provided, the study intervention will be discontinued, and the research ethics committee will be notified within 24 hours with a detailed report outlining the nature of the event, actions taken, and outcomes. The participant will be closely monitored until the serious AEs are adequately managed. The study team will conduct a thorough review of the severe AEs to determine whether modifications to the study protocol are necessary to prevent recurrence.
Auditing	23	Frequency and procedures for auditing trial conduct, if any, and whether the process will be independent from investigators and the sponsor<br>The Research Ethics Committee of the Hospital of Chengdu University of Traditional Chinese Medicine, comprising six renowned experts from various fields, conducts audits every six months to monitor trial performance and safety. This committee operates independently of the sponsors and research team, with no competing interests involved.
**Ethics and dissemination**		
Research ethics approval	24	Plans for seeking REC/IRB approval<br>This study protocol has obtained ethical approval from the Research Ethics Committee of the Hospital of Chengdu University of Traditional Chinese Medicine (Approval number: 2024KL-113).
Protocol amendments	25	Plans for communicating important protocol modifications (e.g., changes to eligibility criteria, outcomes,

		analyses) to relevant parties (e.g., investigators, RECs/IRBs, trial participants, trial registries, journals, regulators)<br>Any changes will require approval from both the Research Ethics Committee of the Hospital of Chengdu University of Traditional Chinese Medicine and the International Traditional Medicine Clinical Trial Registry.
Consent or assent	26a	Who will obtain informed consent or assent from potential trial participants or authorized surrogates, and how (see item 32)<br>Pengxuan Gu, Yunjing Jia, and Yuanzhen Mi will obtain informed consent from potential trial participants.
	26b	Additional consent provisions for collection and use of participant data and biological specimens in ancillary studies, if applicable<br>Not applicable for this trial.
Confidentiality	27	How personal information about potential and enrolled participants will be collected, shared, and maintained in order to protect confidentiality before, during, and after the trial<br>Data collection will be conducted using paper CRFs to systematically document study-related information. All paper records will be securely stored in locked cabinets under the supervision of authorized personnel. Electronic data entry will be managed through a double-entry system, conducted by two independent and experienced staff members. Data will be stored in an encrypted research database, accessible only to authorized research personnel. Strict measures will be implemented to ensure data confidentiality and prevent unauthorized access or breaches.

Declaration of interests	28	Financial and other competing interests for principal investigators for the overall trial and each study site<br>None.
Access to data	29	Statement of who will have access to the final trial data set, and disclosure of contractual agreements that limit such access for investigators<br>Qi Xiao, Ziliang Wu, Bing Lin, and Zhonglin Zhang will be responsible for the data and will have access to the final data set.
Ancillary and post-trial care	30	Provisions, if any, for ancillary and post-trial care, and for compensation to those who suffer harm from trial participation<br>Not applicable.
Dissemination policy	31a	Plans for investigators and sponsor to communicate trial results to participants, health care professionals, the public, and other relevant groups (e.g., via publication, reporting in results databases, or other data-sharing arrangements), including any publication restrictions<br>Upon reasonable request, the data sets generated and/or analyzed can be obtained from the corresponding authors. The results of this study will be published in open-access and peer-reviewed journals.
	31b	Authorship eligibility guidelines and any intended use of professional writers<br>We haven’t used such a service.
	31c	Plans, if any, for granting public access to the full protocol, participant-level data set, and statistical code

		This was already mentioned in 18a and 31a.
**Appendices**		
Informed consent materials	32	Model consent form and other related documentation given to participants and authorized surrogates<br>The model consent form and other relevant documentation provided to participants and authorized surrogates are available from the corresponding author upon reasonable request.
Biological specimens	33	Plans for collection, laboratory evaluation, and storage of biological specimens for genetic or molecular analysis in the current trial and for future use in ancillary studies, if applicable<br>Blood and fecal samples from participants will be collected and stored at -80°C for final analysis.
