# Supplementary material for: Efficacy and mechanism of long-snake moxibustion for treating insomnia in breast cancer survivors: study protocol for a randomized controlled trial
Source: Front Neurol. 2025 Apr 30;16:1524412. doi: 10.3389/fneur.2025.1524412 (PMC12074938; doi:10.3389/fneur.2025.1524412)
Supplement: Supplementary file 2 [file Data_Sheet_2.pdf]

The acupuncture points involved in the treatment area.

Acupoint name	International Code	Belonging to	location
Yaoshu	GV2	Points of Governor Vessel	In the sacral region, at the sacral canal hiatus, along the posterior midline.
Yaoyangguan	GV3	Points of Governor Vessel	In the spinal region, in the depression below the spinous process of the fourth lumbar vertebra, along the posterior midline.
Mingmen	GV4	Points of Governor Vessel	In the spinal region, in the depression below the spinous process of the second lumbar vertebra, along the posterior midline.
Xuanshu	GV5	Points of Governor Vessel	In the spinal region, in the depression below the spinous process of the first lumbar vertebra, along the posterior midline.
Jizhong	GV6	Points of Governor Vessel	In the spinal region, in the depression below the spinous process of the 11th thoracic vertebra, along the posterior midline.
Zhongshu	GV7	Points of Governor Vessel	In the spinal region, in the depression below the spinous process of the 10th thoracic vertebra, along the posterior midline.
Jinsuo	GV8	Points of Governor Vessel	1.5 cun below the spinous process of the seventh thoracic vertebra, along the midline of the back.
Zhiyang	GV9	Points of Governor Vessel	1.5 cun below the spinous process of the ninth thoracic vertebra, along the midline of the back.
Lingtai	GV10	Points of Governor Vessel	1.5 cun below the spinous process of the tenth thoracic vertebra, along the midline of the back.
Shendao	GV11	Points of Governor Vessel	In the spinal region, in the depression below the spinous process of the first lumbar vertebra, along the posterior midline.
Shenzhu	GV12	Points of Governor Vessel	In the spinal region, in the depression below the spinous process of the 9th thoracic vertebra, along the posterior midline.
Taodao	GV13	Points of Governor Vessel	In the spinal region, in the depression below the spinous process of the 1st thoracic vertebra, along the posterior midline.
Dazhui	GV14	Points of Governor Vessel	In the spinal region, in the depression below the spinous process of the 7th cervical vertebra, along the posterior midline.
Dazhu	BL11	Points of Bladder Meridian	In the thoracic region, 1.5 cun lateral to the posterior midline, below the spinous process of the 1st thoracic vertebra.

Fengmen	BL12	Points of Bladder Meridian	In the thoracic region, 1.5 cun lateral to the posterior midline, below the spinous process of the 2nd thoracic vertebra.
Feishu	BL13	Points of Bladder Meridian	In the back spinal region, 1.5 cun lateral to the posterior midline, below the spinous process of the 3rd thoracic vertebra.
Jueyinshu	BL14	Points of Bladder Meridian	In the back spinal region, 1.5 cun lateral to the posterior midline, below the spinous process of the 4th thoracic vertebra.
Xinshu	BL15	Points of Bladder Meridian	In the back spinal region, 1.5 cun lateral to the posterior midline, below the spinous process of the 5th thoracic vertebra.
Dushu	BL16	Points of Bladder Meridian	In the back spinal region, 1.5 cun lateral to the posterior midline, below the spinous process of the 6th thoracic vertebra.
Geshu	BL17	Points of Bladder Meridian	In the back spinal region, 1.5 cun lateral to the posterior midline, below the spinous process of the 7th thoracic vertebra.
Ganshu	BL18	Points of Bladder Meridian	In the back spinal region, 1.5 cun lateral to the posterior midline, below the spinous process of the 9th thoracic vertebra.
Danshu	BL19	Points of Bladder Meridian	In the back spinal region, 1.5 cun lateral to the posterior midline, below the spinous process of the 10th thoracic vertebra.
Pishu	BL20	Points of Bladder Meridian	In the back spinal region, 1.5 cun lateral to the posterior midline, below the spinous process of the 11th thoracic vertebra.
Weishu	BL21	Points of Bladder Meridian	In the back spinal region, 1.5 cun lateral to the posterior midline, below the spinous process of the 12th thoracic vertebra.
Sanjiaoshu	BL22	Points of Bladder Meridian	In the spinal region, 1.5 cun lateral to the posterior midline, below the spinous process of the 1st lumbar vertebra.
Shenshu	BL23	Points of Bladder Meridian	In the spinal region, 1.5 cun lateral to the posterior midline, below the spinous process of the 2nd lumbar vertebra.
Qihaishu	BL24	Points of Bladder Meridian	In the spinal region, 1.5 cun lateral to the posterior midline, below the spinous process of the 3rd lumbar vertebra.
Dachangshuu	BL25	Points of Bladder Meridian	In the spinal region, 1.5 cun lateral to the posterior midline, below the spinous process of the 4th lumbar vertebra.

Guanyuanshu	BL26	Points of Bladder Meridian	In the spinal region, 1.5 cun lateral to the posterior midline, below the spinous process of the 5th lumbar vertebra.
Xiaochangshu	BL27	Points of Bladder Meridian	In the sacral region, at the level of the first sacral posterior foramen, 1.5 cun lateral to the sacral midline.
Pangguangshu	BL28	Points of Bladder Meridian	In the sacral region, at the level of the second sacral posterior foramen, 1.5 cun lateral to the sacral midline.
Zhonglushu	BL29	Points of Bladder Meridian	In the sacral region, at the level of the third sacral posterior foramen, 1.5 cun lateral to the sacral midline.
Baihuanshu	BL30	Points of Bladder Meridian	In the sacral region, at the level of the fourth sacral posterior foramen, 1.5 cun lateral to the sacral midline.
Shangliao	BL31	Points of Bladder Meridian	In the sacral region, directly in line with the first sacral posterior foramen.
Ciliao	BL32	Points of Bladder Meridian	In the sacral region, directly in line with the second sacral posterior foramen.
Zhongliao	BL33	Points of Bladder Meridian	In the sacral region, directly in line with the third sacral posterior foramen.
Xialiao	BL34	Points of Bladder Meridian	In the sacral region, directly in line with the fourth sacral posterior foramen.
